# Sabotage, Collusion, and Being a Feeder: Towards a New Model of Negative Social Support and Its Impact on Weight Management

**DOI:** 10.1007/s13679-023-00504-5

**Published:** 2023-06-07

**Authors:** Jane Ogden, Sophia Quirke-McFarlane

**Affiliations:** School of Psychology, University of Surrey, Guildford, GU2 7XH Surrey UK

**Keywords:** Obesity management, Social support, Sabotage, Feeder behaviour, Collusion, Bariatric surgery, Dieting

## Abstract

***Purpose of Review*:**

Whilst research indicates the positive impact of social support across a number of health domains, including weight management, not all social support is beneficial.

***Recent Findings*:**

This paper reviews the evidence for both positive and negative social support in the context of behavioural interventions and surgery for obesity. It then presents a new model of negative social support focusing on sabotage (‘active and intentional undermining of another person’s weight goals’), feeding behaviour (‘explicit over feeding of someone when they are not hungry or wishing not to eat’), and collusion (‘passive and benign negative social support to avoid conflict’) which can be conceptualised within the context of relationships as systems and the mechanisms of homeostasis.

***Summary*:**

There is increasing evidence for the negative impact of social support. This new model could form the basis of further research and the development of interventions for family, friends, and partners to maximise weight loss outcomes.

## Introduction

Social support plays a key role across a number of physical health domains. Social support is typically conceptualised as positive and most research indicates a positive impact of social support on health outcomes. This paper will first briefly review this vast literature exploring the positive impact of social support on health. Next, it will describe evidence for the benefits of social support for weight management for patients engaging in behaviour change interventions and bariatric surgery. Not all social support is positive, however, and this paper will therefore explore the ways in which social support can be detrimental to a person’s weight management attempts. Finally, it will propose a new model of negative social support with a focus on sabotage, feeder behaviour, and collusion which can be understood within a systems approach to relationships and the central role of homeostasis.

## Defining Social Support

Social support has been defined in a number of ways. For example, Cohen and Wills [1] differentiated between esteem support (which increases self-esteem), informational support (giving advice), companionship (through shared activities), and instrumental support (physical help), whilst Lett et al. [2] differentiated between structural support (contact with a network) and functional support (perceived benefit of this network). Furthermore, Sarason et al. [3] focused on both the number of friends available to offer support and the satisfaction with this support. More simply, Wallston et al. [4] considered social support as relating to perceived comfort, caring, esteem, or help from others. In 2020, Bavik et al. [5••] synthesised findings across a number of disciplines from more than 4500 studies and concluded that social support functions through four dynamic roles: as a positivity catalyst, as a positivity enhancer, as a negativity buffer, and as a negativity exacerbator. Therefore, in general, social support is considered to have a beneficial impact upon the individual either through a direct pathway as the presence of social support is itself beneficial or via an indirect pathway with social support acting as a buffer against external stressors.

## The Positive Impact of Social Support on Health Outcomes

In line with this positive perspective, research shows the beneficial impact of social support across a wide range of health outcomes. For example, social support predicts changes in health-related behaviours such as exercise, diet, smoking, contraceptive use, and safer sex practices [6–10]. It is also linked to help-seeking behaviour at the early stages of illness onset [11, 12] and adaptation, adjustment, and quality of life as an illness develops [13–15]. Furthermore, social support and the absence of it in the form of social isolation and loneliness has also been linked with positive outcomes for the management of stress, pain, and chronic conditions such as diabetes, coronary heart disease (CHD), asthma, and cancer [16–18].

Given the benefits of social support for a range of health outcomes, research has also explored the impact of social support in the context of obesity management, and in general, a similar pattern of results is apparent. For example, from their review of the National Weight Control Registry in the USA, Wing and colleagues [19, 20] highlighted a role for social support in weight loss sustained up to 5 years and in their conceptual review of the literature. Elfhag and Rössner [21] also described a positive role for social support on weight maintenance following intentional weight loss by at least 6 months. Furthermore, whilst the systematic review by Varkevisser et al. [22•] identified the absence of high-quality studies which included measures of social support, their search identified six studies that showed an association between increased social support and improved weight maintenance following non-surgical interventions for obesity. Likewise, Street and Avenell [23] concluded from their systematic review that group-based interventions were more effective than individual-based interventions for weight loss by at least 12 months follow-up potentially due to the benefits of social support derived from being part of a group.

Social support has also been linked with outcomes following bariatric surgery. For example, in 2011, Livhits et al. [24] concluded from their systematic review that attendance at post bariatric support groups was predictive of weight loss success following bariatric surgery. Similarly, Athanasiadis et al. [25•] carried out a systematic review of weight regain following Roux-en-Y gastric bypass and sleeve gastrectomy and reported that greater social support was associated with reduced weight regain. Furthermore, Conceicão et al. [26•] concluded from their study that greater social support was associated with lower depression and emotional eating, reduced weight and shape concerns, and greater weight loss in pre- and post-surgery groups. Likewise, Tymoszuk et al. [27] concluded from their prospective study that pre-surgery social support defined in terms of received emotional and practical support and contact with friends and family predicted greater weight loss post bariatric surgery at 3, 12, and 24 months. In a similar vein, much qualitative research has also explored the ways in which patients undergoing weight management interventions experience social support from others and highlights the beneficial impact of support from family, friends, partners, and health care professionals. For example, Wallwork et al. [28] described how their participants offered emotional, physical, practical, and monetary support to their partners undergoing bariatric surgery and Pories et al. [29] described how partners can offer support through reminding their partner to take vitamin supplements. Likewise, both Pories et al. [29] and Woodard et al. [30] described how partners often change their own eating behaviours to support their spouse following surgery described by Pories et al.’s [29] as a “joint effort”, “a team effort”, and/or “a joint journey” (pg. 58).

## The Negative Side to Social Support

Social support therefore seems to have a positive impact on health outcomes across a range of health domains and predicts weight loss, weight maintenance, and improved well-being in the context of obesity management. Increasingly, however, research indicates a more problematic side to social support and indicates that not all forms of support are beneficial. For example, in the context of health-related behaviours, support can lead to coercion and pressure to perform unhealthy behaviours such as unsafe sex [31–33] and drug use [34]. Furthermore, it can lead to delayed help seeking if members of a social network normalise and minimise the severity of symptoms [11] and can result in the exacerbation of a chronic condition if family or partners encourage dependency or passivity which can promote an illness identity and facilitate secondary gains from being ill [35, 36]. In the context of obesity management, research has also pointed to a more negative role for social support. For example, interviews with patients post bariatric surgery indicate that support can be less than optimal with Tolvanen et al. [37] describing how friends and family can be discouraging and at times stigmatising. Likewise, Gerac, Brunt, and Marihart [38] described how patients receive comments that can be hurtful and critical, and Ficaro [39] highlighted how daughters of mothers who have lost weight can feel challenged by this change. Furthermore, Whale, Gillison, and Smith [40] detailed a range of ways in which negative aspects of social support can undermine attempts at weight management particularly if friends feel threatened by the weight loss of others.

Social support may therefore be less than optimal and can have a negative impact on health outcomes, specifically weight management following either behavioural interventions or bariatric surgery. Some forms of this negative social support take the form of the absence of positive social support and can be considered more passive such as the absence of emotional support through not listening to how someone is feeling; the absence of practical support by not being able to take on childcare responsibilities when a partner has hospital visits; or the absence of informational support due to not learning about what is needed to make weight management interventions more successful. In contrast, however, some research points to a more active version of negative social support whereby there is some degree of intentionality behind a person’s actions. To date, evidence highlights three forms of such intentional negative social support which can be conceptualised as sabotage, being a feeder and collusion. These will now be discussed.

## The Act of Sabotage

The act of sabotage relates to the intention to undermine a person’s actions and can be seen across the literature exploring social support for weight management. For example, research exploring the experiences of those attempting to lose weight through behavioural interventions illustrates how these attempts are sometimes undermined by friends and family members who sabotage attempts to lose weight [40–47]. Likewise, research shows a similar pattern for patients post bariatric surgery with family and friends having a negative impact on their degree of success [43]. Much research has also explored the negative impact of intimate partners and illustrates how negative support within couples can undermine both weight loss and weight loss maintenance after both behavioural interventions [41, 48–52] and surgery [28, 43, 48, 49, 53]. Research also highlights the types of sabotage that can occur and indicates a negative impact of social support on health-related behaviours. This includes sabotaging attempts to change eating patterns via processes such as discouraging healthy eating and putting up barriers to attending support groups [40, 43, 48, 49] as well as undermining efforts to increase physical activity through refusing to go for walks or highlighting the cost of a gym membership [45–50, 53]. Furthermore, research also highlights the ways in which forms of sabotage can undermine an individual’s confidence and self-esteem and lower their mood through criticism and hurtful comments [40, 44, 48, 49]. Whilst much of this research specifically uses the term sabotage, either whilst quoting directly from participants or describing the data [45, 46, 50, 53], other studies imply sabotage in their analysis [28, 47].

## Being a Feeder

Sabotage is therefore one form of negative social support that can undermine weight loss attempts. A key part of sabotage relates to eating behaviour, and some research has addressed the explicit and sometimes deliberate provision of food even when the other person is not hungry or trying to eat less which has been called ‘Being a Feeder’ [54••]. Research indicates that people feed others for many reasons including waste avoidance [55], as a sign of family love [56–58]; as a sign of wealth and status [59, 60]; and a marker of power and control [61]. In line with this, Ogden, Cheung, and Stewart [62•] developed a new quantitative measurement tool to assess both the motivations behind feeder behaviour and the behaviour itself which consisted of six motivational subscales and one subscale to measure feeder behaviour. The motivation subscales were as follows: feeding for love (e.g. ‘because I love them’); feeding for waste avoidance (e.g. ‘I don’t like to waste food’); to avoid hunger (e.g. ‘because people shouldn’t go hungry’); to offload food (e.g. ‘because I can’t finish my food’); to show good manners (e.g. ‘because it is polite’); and as a sign of status (e.g. ‘because I want to show how much I have to offer’). The feeder behaviour subscale involved items such as ‘offering people when they are not hungry’. Findings showed that the best motivational predictors of feeder behaviour were love, offloading, manners, and status and that feeder behaviour correlated with measures of restrained eating, external eating, and emotional eating [54••, 62•]. In addition, analysis within intimate relationships indicated that feeder behaviour within couples may not only function in a reciprocal way with each partner feeding the other equally but also in a more linear ways with one partner’s behaviour impacting directly upon their partner [62•]. In the context of weight management, feeder behaviour, not only by partners but also family and friends, could play key role in the degree of weight loss and weight loss maintenance after either behavioural or surgical interventions.

## Collusion

Intentional negative social support can therefore take the form of sabotage involving actively undermining a person’s attempts at weight loss, which may include being a feeder by offering food when the other person does not want to eat. There is, however, a third form of negative social support which happens in a more benign way and involves a degree of collusion. The notion of collusion has been defined, observed, and evaluated as a core part of communication between individuals as a means to maintain conversation and avoid conflict [63, 64]. It also finds reflection in the phrase ‘killing with kindness’ which has been used across disciplines including literature, drama, veterinary medicine, and international aid as well as in the context of obesity [65–68]. Collusion has been used within a therapeutic setting to describe the shared space between therapist and client and a mechanism to avoid disillusionment [69] and has been described within the interactions between therapists and rape survivors [70], patients with suicidal ideation [71], and within marital therapy [72]. It has also been described as a mechanism to reinforce gender stereotypes with one gender presenting themselves in a way that colludes with the stereotype of the other gender [73]. Furthermore, it has been used extensively to describe the dynamic between clinicians and patients receiving end of life care in the context of conversations about dying [74–77]. In the context of weight management, research exploring interactions between health care professionals (HCPs) and patients indicates collusion in their consultations. For example, Atkinson and McNamara [78] interviewed 15 women postnatally who had a body mass index (BMI) > 30 and described how their consultations during pregnancy involved unconscious collusion to ‘navigate or even avoid the issue of obesity’. Likewise, Natvik et al. [79] used dyadic data of patients and HCPs and explored aspects of collusion in consultations post bariatric surgery. Furthermore, whilst not directly labelling negative social support as collusion, several studies highlight ways in which family, friends, and partners collude with those trying to lose weight through ‘going along’ with their behaviour when it is not in line with their weight loss goals [41, 47, 52, 80]. Collusion is less intentional than sabotage or feeder behaviour and often seems to reflect kindness, friendship, and support, but in line with these, more active forms of negative social support also function to undermine weight management goals.

## A New Model of Negative Social Support

**Fig. 1:**
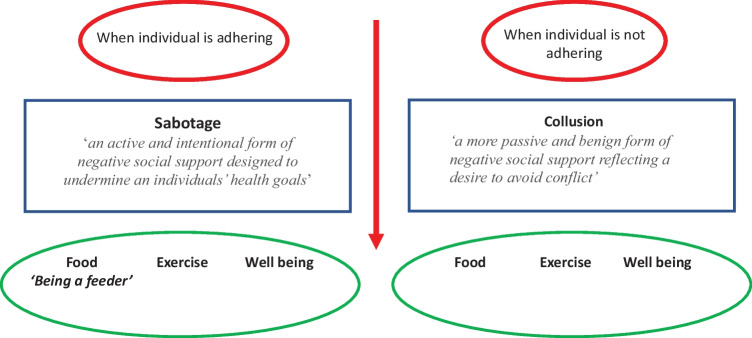
Negative social support: the role of sabotage, collusion, and being a feeder

Therefore, whilst much social support research illustrates a positive impact on health outcomes, there are also versions of negative social support which can have a detrimental impact on the health of others. This review has highlighted a more intentional version of negative social support in the form of sabotage and a more passive and benign version in the form of collusion. Both sabotage and collusion can undermine an individual’s attempts to lose weight through their diet, exercise, or well-being. Furthermore, feeder behaviour has been identified as a specific form of sabotage when this sabotage act is aimed at food and encourages an individual to eat more than they would prefer. From this analysis of the literature, sabotage can be defined as ‘an active and intentional form of negative social support designed to undermine an individuals’ health goals’; collusion can be defined ‘a more passive and benign form of negative social support reflecting a desire to avoid conflict’; and being a feeder can be defined as ‘the explicit over feeding of someone else even when they are not hungry or do not express a desire to eat’. To date, it remains unclear how prevalent these different forms of negative social support are and the extent to which they impact upon an individual’s weight management attempts. Furthermore, it is unclear whether the presence of negative social support is better or worse than having no social support at all. These forms of negative social support are illustrated in Fig. 1.

## Social Support as Part of a System

Research therefore indicates that when an individual tries to lose weight, they may be confronted by negative social support in the form of sabotage, collusion, or feeding behaviour by those closest to them. This process can be understood in the context of systems’ theory and the notion of homeostasis [81–84]. From this perspective, relationships are conceptualised as existing within a dynamic system with its members motivated to maintain an equilibrium and the status quo. As Minuchin argued in 1985, this system is ‘an error activated process by which behaviour departing from the expected range of a family’s patterns is controlled via corrective feedback loops’ [83] (p. 290). Therefore, any change to the status quo is managed by the mechanisms of homeostasis enacted to bring the system back to what is familiar and safe. In the context of social support, some components of positive support may facilitate change and encourage the individual to lose weight and bring about a change in the system. This finds reflection in research exploring epiphanies and teachable moments and how successful weight loss and weight loss maintenance can occur given the right set of sustaining conditions [85, 86]. In contrast, however, negative social support processes such as sabotage, collusion, and feeder behaviour illustrate homeostatic mechanisms which re-establish the status quo in the face of changes threatened by weight loss and the newly attempted behaviours associated with it.

## Conclusion

Much research to date has focused on the positive aspects of social support across a range of health outcomes including obesity management. Increasing evidence, however, also points to a more detrimental version of social support including sabotage, collusion, and being a feeder. Further research is needed to explore the prevalence of these forms of negative support and the extent to which they impact upon an individual throughout their weight loss journey whether it be through behavioural interventions, surgery, or medication taking. Furthermore, an understanding of negative social support may help explain the higher rates of relationship change, including divorce, separation, and the onset of new relationships after bariatric surgery, particularly for those who show higher levels of weight loss [87]. Such research could form the basis of interventions targeting family, friends, or partners as a means to maximise their positive impact on weight loss and weight loss maintenance and minimise the detrimental consequences of any forms of negative social support whether intentional and explicit or more benign and implicit. This paper has therefore presented a new model of the more negative aspects of social support which involve more intentional processes which undermine an individual’s weight management attempts. These can be understood as a mechanism of homeostasis and could provide the basis for future research and interventions to support patients as they navigate the impact their attempts to lose weight have on their relationships surrounding them.
